# Restless legs syndrome and quality of sleep in patients with glomerulopathy

**DOI:** 10.1186/1471-2369-14-113

**Published:** 2013-05-28

**Authors:** Alexandre Braga Libório, João Paulo Lima Santos, Natália Feitosa Arraes Minete, Cecília Alencar de Diógenes, Luiza de Andrade Braga Farias, Veralice Meireles Sales de Bruin

**Affiliations:** 1 Department of Clinical Medicine – Faculdade de Medicina, Universidade Federal do Ceará, Avenida Abolição no. 4043 Ap. 1203 Edifício Jangada, Mucuripe, CEP 60.165-082, Fortaleza, Ceará, Brazil; 2 Medical Course, Universidade de Fortaleza, Fortaleza, Ceará, Brazil; 3 Nephrology Department, Hospital Geral de Fortaleza, Fortaleza, Ceará, Brazil; 4 Post-graduate Program, Universidade de Fortaleza, Fortaleza, Ceará, Brazil

**Keywords:** Sleep quality, Nephrotic syndrome, Proteinuria

## Abstract

**Background:**

Despite a confirmed association between restless legs syndrome (RLS) and end-stage renal disease (ESRD), there is no study on patients presenting with nephrotic syndrome (NS). To investigate the frequency of RLS and poor quality sleep in NS-patients secondary to primary glomerulopathy with nearly normal glomerular filtration rate (GFR) and its associated factors.

**Methods:**

Patients with NS, defined as 24 h-urine protein greater than 3.5 g/1.73 m^2^ and hypoalbuminemia, (n = 99, 53 women) and a mean age of 36±11 years were studied. Age and sex-matched controls were used to compare RLS and poor sleep quality prevalence. Standardized RLS questionnaire formulated by the International Restless Legs Syndrome and Pittsburgh Sleep Quality Index (PSQI) were used.

**Results:**

RLS was more frequent in NS-patients than in controls (22.8 vs. 4.0%, p = 0.01). Mean time since diagnosis (52.2±34.1 vs. 28.6±22.5 months, p < 0.01) and 24 h-proteinuria (3.7±1.3 vs. 2.6±0.6 g/1.73 m^2^, p = 0.001) were greater in NS-patients with RLS those not presenting RLS. Association between RLS with 24 h-proteinuria [OR = 2.31; p = 0.007; 95% CI 1.87-2.89] and time since diagnosis [OR = 1.10; p = 0.003; CI = 1.02-1.39] were identified even after controlling for age, GFR and diabetes. Sleep quality was poor in NS-patients than in controls (mean PSQI score 7.35±3.7 vs. 5.2±3.0, p = 0.003). In NS-patients, only RLS was associated with poor sleep quality (OR = 1.20; p = 0.004).

**Conclusion:**

Poor quality sleep and RLS are frequent in NS-patients without ESRD. Pathophysiology of this association must be further investigated.

## Background

Glomerulopathy is a group of diseases that affect mainly young adults between 20–40 years old [1]. Nephrotic syndrome is one major presentation of glomerulopathies and patients generally present with important edema, lipid alterations, hypoalbuminemia, and possible loss of renal function. Also, inflammation, oxidative stress and endothelial dysfunction are associated-features of nephrotic syndrome [2].

The diagnosis of RLS is clinically based and requires the presentation of all of the following four main symptoms: (1) an urge to move the legs, usually accompanied by unpleasant sensations; (2) precipitation of symptoms by rest and inactivity; (3) symptom relief by movement; and (4) worsening appearance in the evening or at night [3].

Restless legs syndrome, a sensory-motor neurological disorder, can occur idiopathic or in association with other clinical conditions [4]. It is a common disorder in hemodialysis patients frequently leading to poor quality sleep and daytime somnolence [5]. Restless legs syndrome is a common disorder in patients with advanced chronic kidney disease (CKD) and hemodialysis patients frequently leading to poor quality sleep and daytime somnolence [5-7]. However, there is no study specifically in patients with NS-associated primary glomerulopathy. The aim of the present study was to estimate the prevalence of RLS and sleep quality in NS-patients due primary glomerulopathy. Moreover, we intended to evaluate NS-features associated with sleep disorders.

## Methods

### Study design

This was a cross-sectional study of patients submitted to renal biopsy due NS at a reference university hospital of Brazil (Hospital Geral de Fortaleza) from October/2010 to December/2011. Demographic data, habits and comorbidities were recorded using specific questionnaires answered concurrently in a face-to-face interview performed by general physicians after adequate training. Nephrotic syndrome was defined as 24 h-urine protein greater than 3.5 g/1.73 m^2^ associated with edema, hypoalbuminemia (less than 3.0 g/dL) and lipid alteration (low-density lipoprotein cholesterol – LDL > 130 mg/dL and/or triglycerides > 300 mg/dL). Only patients presenting primary minimal lesions (ML), focal segmental glomerulosclerosis (FSGS), Membranous Nephropathy (MN), IgA nephropathy (IgAN) and membranoproliferative glomerulonephritis (MPGN) confirmed with kidney biopsy were included in this study. Diagnosis was performed using light microscopy and immunofluorescence studies. Electron microscopy is not performed routinely at our service. Patients with any evidence of secondary glomerulopathy or advanced CKD (GFR less than 15 mL/min/1.73 m^2^) were excluded. Age and sex-matched healthy subjects were selected from the community as control group. The study protocol was approved by the Research Ethics Committee of Hospital Geral de Fortaleza and written informed consent was obtained in all cases.

#### Measurements

Restless legs syndrome was investigated according to the criteria of the International RLS Study Group (IRLSG) [3]. This scale has been adapted for Brazilian patients [8]. This questionnaire-based RLS diagnosis requires answers consistently indicating presence of all four of the basic RLS diagnostic criteria [9], i.e.: (1) A compelling urge to move the legs usually accompanied by uncomfortable feelings in the legs that must be, (2) engendered or exacerbated by rest (sitting or lying still), (3) relieved by movement, and (4) worse in the evening and night than the morning except for very severe cases when it occurs at all times of the day. To exclude RLS “mimics” [10], a unique neurologist evaluated patients with RLS diagnosed by questionnaire. Also, patients under statin therapy were routinely screened for rhabdomyolysis with creatinine kinase measurement.

Sleep quality was evaluated by the Pittsburgh Sleep Quality Index (PSQI) [11]. This scale has seven components, each one dealing with a major aspect of sleep. Individuals with a PSQI score > 6 were considered poor sleepers.

All patients included in the study were being currently treated with angiotensin-converting enzyme inhibitors (ACE inhibitor) or AT1- receptor blocker (AT1R-blocker) in the absence of formal contraindications. In case of steroid dependency or resistance, in ML and FSGS, the primary treatment was cyclosporine (CsA). MN was treated primarily with CsA or steroids plus cyclophosphamide according to baseline renal function. IgAN presenting with nephrotic syndrome was treated preferentially with steroids and MPGN patients received no immunosuppressive treatment.

#### Laboratory data and definitions

Medical records were retrieved to assess laboratory data at the kidney biopsy time and the last evaluation prior to study inclusion. Laboratory data included an assessment of serum creatinine, total blood count, serum albumin, total cholesterol, LDL cholesterol, High-density lipoprotein (HDL) cholesterol, triglycerides, ferritin, serum iron, 24 h-urine protein excretion rate (24 h-proteinuria) and urinalysis. Serum creatinine was measured using a Jaffe alkaline picrate assay. Serum albumin was measured using bromocresol green colorimetric method. Total cholesterol was measured using enzymatic method; HDL-cholesterol by catalase inhibition; LDL-cholesterol by Friedewald equation and triglycerides by enzymatic method. Serum iron dosage was performed by ferrozine reagent method and ferritin by turbidimetry.

Immunosuppressive treatment was considered only when it was being used in the month anteceding the interview. Edema was considered when it was present in the last clinical evaluation. Hypertension was defined as blood pressure above 140 × 90 mmHg at three or more medical evaluation or a positive high blood pressure history under regular treatment. Current smoker was considered when patients had smoked at last three months. Hematuria was considered as more than five red blood cells per high-power microscopic field in two urine samples. Total remission was considered when 24 h-proteinuria was less than 500 mg; partial remission when there was a reduction greater than 50% of initial 24 h-proteinuria and it was less than 3.5 g/24 h/1.73 m^2^. Estimated GFR (eGFR) was calculated using simplified MDRD equation.

#### Statistical analysis

Descriptive statistics are expressed as mean ± SD or absolute numbers, as appropriate. Student’s *t*-test was applied to compare continuous variables with equality of variance and normal distribution. Categorical data were compared using the chi-square or Fischer’s test when appropriate. Logistic regression analysis was performed to examine associations between variables and the presence of RLS. We forced in the model factors associated with RLS in previous studies (age, eGFR, ferritin levels and diabetes). The statistical analysis was performed using SPSS 19.0.

## Results

From 142 patients submitted to renal biopsy during study period, 116 had NS due primary glomerulopathy. From these, 108 were invited to study participation and nine refused. Ninety-nine patients (53 female) were included in this study. Subject characteristics are given in Table 1. Mean age was 36.2±11.8 years. Main histological diagnosis was ML/FSGS (n = 53) and MN (n = 29). Forty-three patients were receiving steroid drugs and nineteen received cyclosporine-based therapy. Thirty-eight patients were in total remission, 26 were in partial remission and 35 did not achieve remission. Significant edema was present in 24 patients (25.5%) and 33 (35.1%) had associated arterial hypertension. The mean eGFR was 61±29 mL/min. None of the patients had ESRD (eGFR < 15 mL/min) and the great majority (n = 79) had eGFR higher than 60 mL/min. The mean time of glomerulopathy diagnosis was 33.6±26 months.

**Table 1:** Clinical and laboratory characteristics of all patients and according RLS presence

	**All glomerulopathy patients (n = 99)**	**Glomerulopathy patients with RLS (n = 18)**	**Glomerulopathy patients with no RLS (n = 81)**	**p**
Age (years)	36.2±11.8	34.4±9.1	36.5±14.8	0.359
Gender (M/F)	46/53			
Renal biopsy diagnosis				
*FSGS/ML*				
*MN*	53	9/18	44/81	0.417
*IgAN*	29	5/18	24/81	
*MPGN*	9	2/18	7/81	
	8	2/18	6/81	
Disease duration (months)	33.6±26.0	52±34	28±22	0.006
Arterial Hypertension	40	7/18	33/81	0.853
Diabetes	6	1/18	5/81	0.946
Current smoker	7/99	2/18	5/81	0.614
Diagnosed Cardiovascular Disease	4/99	1/18	3/81	0.563
Presence of edema	31	7/18	24/81	0.443
Mean eGFR (mL/min/1.73 m^2^)	61.2±29.4	64.1±27.6	60.9±30.5	0.872
CKD stage				
*stage 1*	55	8/18	47/81	0.406
*stage 2*	24	7/18	17/81	
*stage 3*	16	2/18	14/81	
*stage 4*	04	1/18	3/81	
Statin therapy	21/99	3/18	18/81	0.756
Antideperessant/neuroleptic therapy	4/99	1/18	3/81	0.558
ACE inhibitor/AT1R blocker	62	13/18	49/81	0.662
Steroid therapy	43	10/18	33/81	0.485
Cyclosporine therapy	19	3/18	16/81	0.802
Cyclophosphamide therapy	03	0/18	3/81	1.0
Hemoglobin (g/dL)	12.3±0.9	12.1±0.8	12.4±1.0	0.878
Serum Ferritin (μg/L)	56.7 ± 31.0	48.6±29.1	58.8±33.4	0.683
Last serum albumin (g/dL)	3.6±0.9	3.4±1.2	3.7±0.9	0.196
Last total cholesterol (mg/dL)	221.2±98.9	239.0±84.3	218.6±46.4	0.258
Last triglycerides (mg/dL)	146.2±58.3	164.1±69.0	142.2±42.4	0.389
Proteinuria at diagnosis (g/24 h/1.73 m^2^)	8.6±3.8	9.1±4.1	8.5±3.7	0.395
Last proteinuria (g/24 h/1.73 m^2^)	2.8±2.2	3.7±1.3	2.6±0.6	0.001
Remission				
Total/Partial	64	10/18	54/81	0.419
No	35	8/18	27/81	
PSQI	7.35±3.7	8.96±3.9	6.99±3.5	0.003

*FSGS*: focal and segmentar glomerulosclerosis; *ML*: minimal lesions; *MN*: membranous Nephropathy; *IgAN*: IgA nephropathy; *MPGN*: membranoproliferative Glomerulonephritis; *CKD*: Chronic Kidney Disease; *ACE*: angiotensin converting enzyme; *AT1R*: angiotensin 1 receptor; *PSQI*: Pittsburgh Sleep Quality Index.

RLS was detected in 18 individuals with glomerulopathy and in 4 sex and age-matched controls with no-previous history of renal disease (22.8 vs. 4.0%, p = 0.01). After univariate analysis, patients with RLS had a greater mean time of diagnosis (52±34 vs. 28±22 months, p < 0.01) and greater 24 h-proteinuria (3.7±1.3 vs. 2.6±0.6 g/1.73 m^2^, p = 0.001) (Table 1). Even after forced adjustment for age, eGFR and diabetes, an association between RLS, time of diagnosis and 24 h-proteinuria was confirmed by logistic regression analysis odds ratio [OR = 1.10; p = 0.003; 95% CI = 1.02-1.39], for each month since diagnosis and 2.31 [95% CI 1.87-2.89], p = 0.007), for each gram of 24 h-proteinuria, respectively – Table 2.

**Table 2:** Association between restless legs syndrome with last 24 h-proteinuria and time of glomerulopathy diagnosis after adjusting for age, estimated glomerular filtration rate and diabetes

**Variable**	**Odds-ratio**	**95% CI**	**P value**
Last 24 h-proteinuria (per each gram)	2.192	1.812-2.741	0.008
Time from diagnosis (per each month)	1.131	1.039-1.475	0.002

After adjusting for age, estimated glomerular filtration rate, serum ferritin and presence of diabetes.

**Figure 1:**
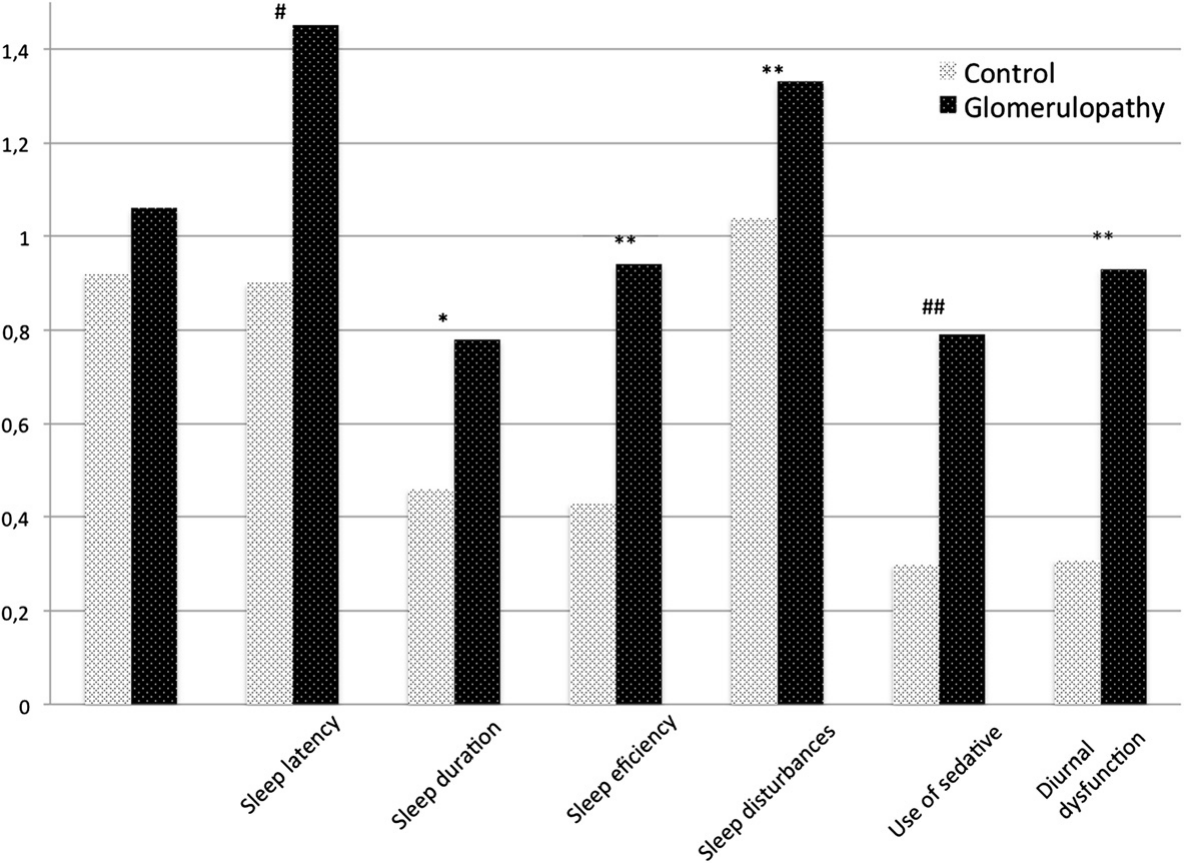
**Study subjects’ quality of sleep components (sleep dimensions) as assessed by the PSQI.** Graph represents mean points in each sleep domain in control (white bar) or glomerulopathy group (black bar). # p = 0.003, * p = 0.04, **p < 0.0001, ## p = 0.02.

Patients with glomerulopathy had poor quality sleep when compared to control group (mean PSQI score 7.35±3.7 vs. 5.20±3.0, p = 0.003). Also, a PSQI score > 6 was more frequent in patients than in controls (52.5 vs. 28.2%, p < 0.001). Cases showed longer sleep latency (p = 0.003), shorter sleep duration (p = 0.04), less sleep efficiency (p < 0.0001), more sleep disturbances (p < 0.0001), more use of sedatives (p = 0.02) and more diurnal dysfunction (p < 0.0001) – Figure 1. Only RLS was associated with a poor sleep quality in GP patients (PSQI > 6) – (OR = 1.20; p = 0.004; CI = 1.05-1.37) after adjustment for age, eGFR and diabetes. There was no association between sleep quality and 24 h-proteinuria or serum albumin levels.

## Discussion

The most important result of this study is show, for the first time, an increased frequency of RLS in NS-patients. Moreover, these patients presented poor quality sleep when compared with an age and sex-matched controls. In view of recent advances in the RLS management, inclusive with new proposed therapies [12,13], the early diagnosis is important to reduce RLS impact on the patients.

Previous studies involving patients with chronic kidney disease (CKD) have shown RLS as highly prevalent and associated with anemia, GFR reduction, serum parathormone and iron status [14,15]. Of importance, in our data, 79/99 of patients had an eGFR greater than 60 mL/min/1.73 and none had ESRD. CKD-related complications such as anemia, uremia, secondary hyperparathyroidism and neuropathy are uncommon in these stages of CKD [16]. Also, it explains why serum ferritin, a known inflammatory marker in CKD patients, was not increased in our sample. This makes possible to evaluate the effects of other clinical features of NS on RLS, such as, edema, hypoalbuminemia, dyslipidemia and urine protein excretion rate.

In this study, RLS was more than four times as likely to be present in patients as in age and sex-matched controls and these results are similar to those described in hemodialysis patients (21%) in another study performed in our region [7]. Overall, RLS is associated with aging [17]: thus, the low age range in the present studied population highlights the importance of RLS in association with glomerulopathy. As expected due to the higher eGFR in these patients, renal function was not associated with RLS. Time of diagnosis and last values of 24 h-proteinuria were the only independently associated with RLS.

Twenty-four hours proteinuria is the main marker of glomerulopathy activity. Generally, higher urine protein excretion is associated with dyslipidemia, inflammation, endothelial dysfunction and renal function decline [2]. All these findings are recognized factors associated with RLS in patients with CKD [18,19]. Due absence of data about inflammation and endothelial function, we cannot speculate about the precise pathophysiological mechanisms underling this association.

A large proportion of GP patients also reported poor quality sleep. No clinical or laboratory parameter related to nephrotic syndrome or its treatment was associated with a PSQI > 6. In contrast, RLS was itself associated with poor sleep quality and this highlights the importance of diagnosing RLS. A recent study has been associated with RLS with cardiovascular disease, especially in patients with CKD [20]. Considering GP are under increased risk of vascular disease [21], mainly due progressive decline in renal function, dyslipidemia, endothelial dysfunction, it is important to diagnose and treat RLS in this population.

## Conclusion

This data is relevant because it describes for the first time a high prevalence of RLS and poor sleep quality in NS patients. Moreover, we have shown that urine protein excretion rate, the main marker of glomerulopathy activity, is independently associated with RLS. Further studies are warranted to investigate the mechanisms underling this association.

## Abbreviations

RLS: Restless legs syndrome; ESRD: End-stage renal disease; NS: Nephrotic syndrome; GFR: Glomerular filtration rate; PSQI: Pittsburgh sleep quality index; CKD: Chronic kidney disease; ML: Minimal lesion; FSGS: Focal and segmental glomerulosclerosis; IgAN: IgA nephropathy; MPGN: Menbranoproliferative glomerulopathy; IRLSG: International restless leg syndrome study group; ACE: Angiotensin converter inhibitor; CsA: Cyclosporine.

## Competing interests

The authors declare that they have no competing interest.

## Authors’ contribution

ABL conceived the study, performed statistical analysis and participated in manuscript writing. JPLS collected data and participated in manuscript revision. NFAM collected data and participated in manuscript revision. CAD collected data and participated in manuscript revision. LABF collected data and participated in manuscript revision. VMSB performed statistical analysis and revised the manuscript writing. All authors read and approved the final manuscript.

## Pre-publication history

The pre-publication history for this paper can be accessed here:

http://www.biomedcentral.com/1471-2369/14/113/prepub
